# Transcatheter mitral and tricuspid interventions—the bigger picture: valvular disease as part of heart failure

**DOI:** 10.3389/fcvm.2023.1091309

**Published:** 2023-05-15

**Authors:** Jonathan Curio, Alessandro Beneduce, Francesco Giannini

**Affiliations:** ^1^ Department of Cardiology, Heart Center Cologne, University of Cologne, Faculty of Medicine and University Hospital, Cologne, Germany; ^2^ Interventional Cardiology Unit, San Raffaele Hospital, Milan, Italy; ^3^ Interventional Cardiology Unit, IRCCA Ospedale Galeazzi Sant'Ambrogio, Milan, Italy

**Keywords:** transcatheter treatment, mitral regurgitation, tricuspid regurgitation, heart failure, valvular disease of the heart

## Abstract

The prevalence of mitral (MR) and tricuspid regurgitation (TR), especially in heart failure (HF) populations, is high. However, the distinct role of atrioventricular valve diseases in HF, whether they are merely indicators of disease status or rather independent contributors in a vicious disease cycle, is still not fully understood. For decades, tricuspid regurgitation (TR) was considered an innocent bystander subsequent to other heart or lung pathologies, thus, not needing dedicated treatment. Recent increasing awareness towards the role of atrioventricular valve diseases has revealed that MR and TR are, in fact, independent predictors of outcome in HF, thus, warranting attention in the HF treatment algorithm. This awareness arose, especially, with the development of minimally invasive transcatheter solutions providing new treatment options, which can also be used for patients considered as having increased surgical risk. However, outcomes of such transcatheter treatments have, in part, been sub-optimal and likely influenced by the status of the concomitant HF disease. Thus, this review aims to summarize data on the current understanding regarding the role of MR and TR in HF, how HF impacts outcomes of transcatheter MR and TR interventions, and how the understanding of this relationship might help to identify patients that benefit most from these therapies, which have proven to be lifesaving in properly selected candidates.

## Introduction

Severe symptomatic mitral (MR) and tricuspid regurgitation (TR) have been identified as independent predictors of mortality (1, 2). Furthermore, patients with significant forms of MR or TR show a significantly increased risk of heart failure (HF) hospitalizations, prolonged hospitalizations, and repetitive re-hospitalizations (3–7). When followed up for at least two years, untreated MR results in HF hospitalization in over 50% of patients, and in patients with untreated TR, over 35% are hospitalized by that time and these HF hospitalizations are independently associated with increased mortality (3, 6). In recent years, this sparked the evolution of novel, less invasive transcatheter treatment approaches, especially as the population of MR and TR patients is often elderly, multi-morbid, and at high risk for surgery (8, 9). A broad range of devices underwent pre-clinical and clinical testing, and several techniques have been established in actual practice (10, 11). Besides other approaches like annuloplasty or valvular replacement, the most prominent and most frequently used treatment modality to date is transcatheter edge-to-edge (TEER) repair of either the mitral (MV) or the tricuspid valve (TV) (12, 13).

**Figure 1:**
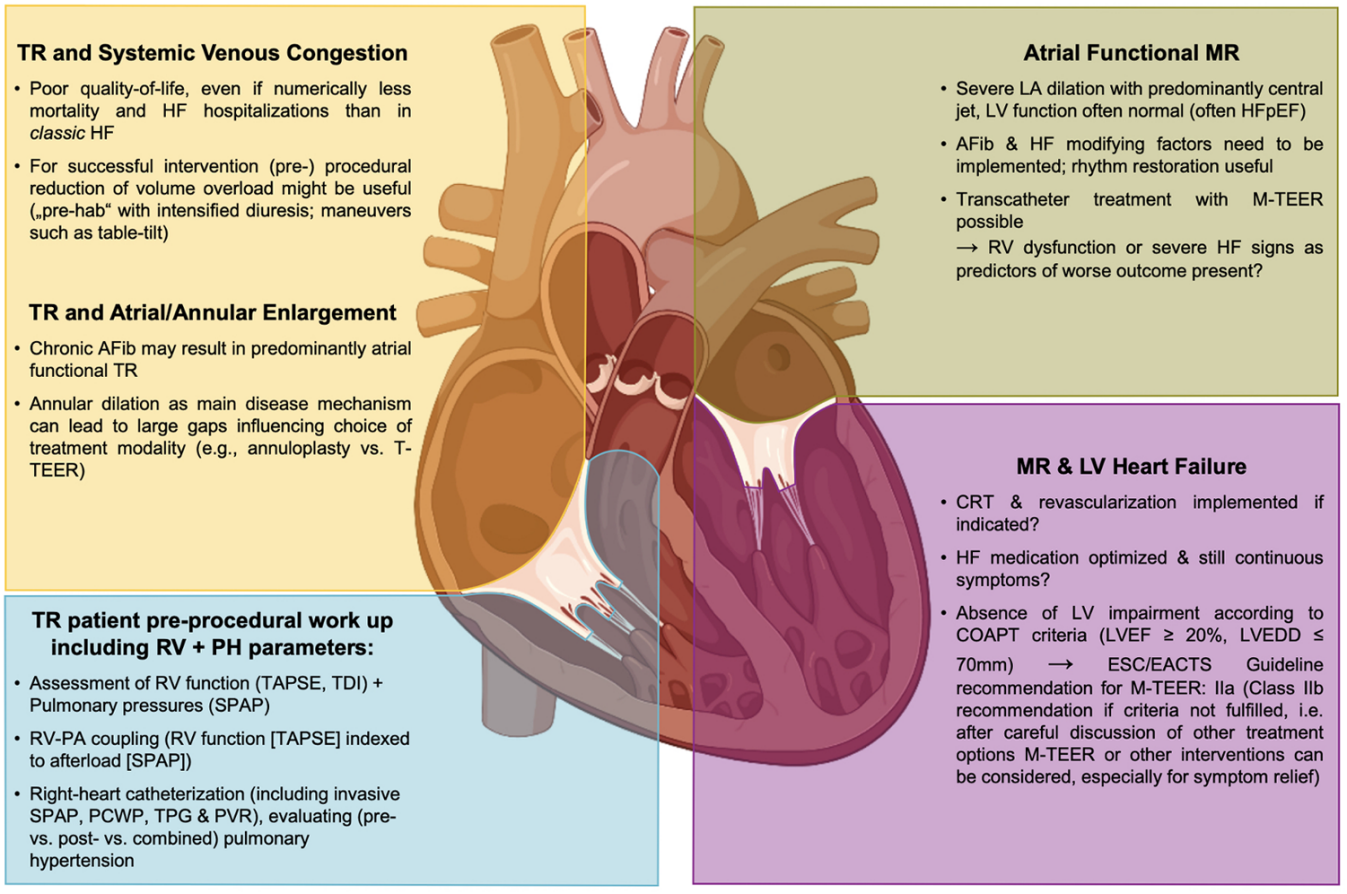
MR and TR in the HF disease conundrum and as part of a systemic disease—implications for interventional therapies. AFib, atrial fibrillation; CRT, cardiac resynchronization therapy; HF, heart failure; HFpEF, heart failure with preserved ejection fraction; LA, left atrium; LV, left ventricle; LVEDD, left ventricular end-diastolic diameter; LVEF, left ventricular ejection fraction; MR, mitral regurgitation; M-TEER, mitral transcatheter edge-to-edge repair; PA, pulmonary artery; PCWP, pulmonary capillary wedge pressure; PVR, pulmonary vascular resistance; RV, right ventricle; SPAP, systolic pulmonary artery pressure; TAPSE, tricuspid annular plane systolic excursion, TDI, tissue Doppler imaging; TPG, trans pulmonary gradient; T-TEER, tricuspid transcatheter edge-to-edge repair, TR, tricuspid regurgitation.

A lot of attention has been paid to outcomes after interventional treatment in patients with secondary forms of MR (SMR) or TR (STR) most often presenting in the setting of chronic HF. For mitral TEER (M-TEER) in patients with HF and reduced ejection fraction (HFrEF), two large randomized trials, namely the COAPT trial and the Mitra-FR trial, have resulted in diverging outcomes. In the COAPT trial, a significant benefit of M-TEER, when added to optimal guideline-directed medical therapy (GDMT), was evident, while in the Mitra-FR trial, the additive effect of interventional treatment was neutral (14, 15). These results initiated ongoing discussions regarding potential explanations for such a divergence. The first agreement has been reached that an assessment of potential M-TEER candidates must not only look at the valvular lesion itself but also has to incorporate a distinct assessment of ventricular function and dimensions, and concomitant HF has to be addressed as a holistic disease entity, in general (Figure 1) (16–18).

Interventional treatment of severe TR, on the other hand, has caught up at a rapid pace in the last years, first using established M-TEER devices in the tricuspid position (T-TEER) but now also utilizing dedicated devices, including several replacement solutions (19–22). The identification of TR as an independent predictor of mortality as well as bad outcomes of isolated TV surgery with high in-hospital mortalities of up to 10% in these patients meant an unmet clinical need that these new devices are now trying to address (1, 23, 24). However, following the historic belief that TR is only secondary to left-sided heart disease and would diminish with treatment, the awareness towards TR still is too little (25). Thus, patients are referred late in their disease course, often presenting multi-morbid and complex chronic HF status (9). In such patients, even though a propensity-matched analysis of the TriValve registry demonstrated a benefit with transcatheter TR treatment compared to GDMT alone, even when treated, rates of 1-year mortality and HF rehospitalization are high (26). Thus, in such cohorts, the delineation between patients benefiting from intervention and those in whom a transcatheter treatment may be futile represents a challenge for heart teams when evaluating patients suffering from persistent HF symptoms and valvular heart disease.

Given this interplay of chronic HF with SMR and STR, this review aims to define the role of these valvular lesions in the HF disease complex and summarize the reported response to transcatheter treatment according to different HF characteristics, and based on this, tries to understand which parameters might be of use to identify those patients most likely to benefit from interventional MR and/or TR treatment.

## Mitral regurgitation

### MR in the context of heart failure

In the European population, MR represents the most common heart valve disease and is the second most common reason for heart valve surgery after aortic stenosis: MR is present in 2% of the overall population, being  ≥  moderate in 2.3% of people ≥65 years, and in 9.3% of people ≥75 years (27, 28). Rossi et al. found that in patients with chronic HF due to non-ischemic or ischemic dilated cardiomyopathy [mean left ventricular (LV)EF: 32% ± 8%], 49% had mild to moderate and 24% had severe SMR (29). Trichon et al. reported that in patients with left ventricular systolic dysfunction (LVEF <40%), any MR was present in 56%, and of these, 30% had severe MR (30). Goliasch et al. identified ≥ moderate MR in 53% of patients in a large HFrEF cohort [median LVEF: 27 (20–35) %] (31). In all these HFrEF cohort studies, MR was independently associated with increased mortality and HF rehospitalization rates. Interestingly, Goliasch et al. found that SMR, especially, is associated with worse outcomes in an intermediate type of HFrEF patients (NYHA class II/III, moderately reduced LVEF of 30%–40%, and NT-proBNP in the second quartile of 871–2,360 pg/ml) (31).

In addition to established SMR definitions, atrial functional MR has been recently discussed as a distinct form of SMR (32–35). Typically, these patients present with long-standing atrial fibrillation or HF with preserved LVEF, leading to atrial enlargement and annular dilation, while ventricular dimensions are without any impairment. Identifying such specific anatomical factors may impact the therapeutic management like patients' rhythm management, or an intervention focusing on aspects of annular dilation may be the preferred treatment.

For any form of SMR, it is important to highlight that its severity may dynamically vary depending on loading conditions (36). Thus, during the work-up of patients who suffer from HF symptoms and show some form of SMR, the additional performance of exercise echocardiography can unmask significant changes in SMR severity, which has been identified as an important prognostic marker of poor outcomes (37, 38). It might be that patients with such dynamic and exercise-induced severe SMR derive particular benefits from a timely intervention; however, it is important to highlight that there is currently no sufficient data regarding transcatheter treatment in this specific subset of patients.

### Outcomes of transcatheter treatment in HF patients

Over half of the patients with severe SMR and HF will not undergo surgery because their disease state has a direct impact on outcomes. This scenario represents an unmet clinical need, potentially addressable with M-TEER and other transcatheter solutions (39).

Two large randomized controlled trials evaluated the role of M-TEER in addition to GDMT in the HFrEF population. In the COAPT trial, patients treated with MitraClip (Abbott Laboratories, Chicago, Illinois, USA) when compared to patients with GDMT alone (device group [*n* = 302] baseline characteristics: LVEF: 31.3 ± 9.1%, left ventricular end-diastolic dimension: 6.2 ± 0.7 cm, left ventricular end-diastolic volume [LVEDV]: 101 ± 34 ml/m^2^, NT-proBNP: 5,174.3 ± 6,566.6 pg/ml; see Table 1) experienced significantly fewer annualized HF hospitalizations within 24 months [35.5% vs. 67.9% in GDMT only; HR = 0.53 (95% CI: 0.4–0.7), *p* < 0.001], and had a significantly lower rate of mortality within 24 months [29.1% vs. 46.1% in GDMT only; HR = 0.62 (95% CI: 0.46–0.82), *p* < 0.001]. This corresponds to the number needed to treat 5.9 patients (95% CI: 3.9–11.7) to prevent one death (14). Conversely, in the randomized controlled Mitra-FR trial, there were no significant differences in the rate of HF hospitalizations at 12 months [48.7% vs. 47.4% in GDMT only; HR = 1.13 (95% CI: 0.81–1.56)] and the rate of death from any cause (24.3% vs. 22.4% in GDMT only; HR = 1.11 [95% CI: 0.69–1.77] between patients treated with MitraClip (device group [*n* = 152] baseline characteristics: LVEF: 33.3 ± 6.5%, LVEDV: 136.2 ± 37.4 ml/m^2^, NT-proBNP: 3,407 (1,948–6,790); see Table 1) and patients receiving GDMT only (15).

**Table 1:** Baseline heart failure characteristics in main transcatheter mitral intervention studies.

	COAPT (*n* = 302) (14)	Mitra-FR (*n* = 152) (15)	EuroSMR (*n* = 1,016) (40)	Cardioband 1 year (*n* = 60) (41)	CHOICE-MI (42)
Treatment	M-TEER (MitraClip)	M-TEER (MitraClip)	M-TEER (MitraClip)	Annuloplasty (Cardioband)	Replacement (10 different devices)
Longest follow-up	3 years	2 years	2 years	1 year	1 year
Mortality last follow-up	42.8%	63.80%	32%%	13%%	28%
**Baseline HF characteristics**					
NYHA class III/IV	57%	63%	89%	87%	87%
LVEF (%)	31.3 ± 9.1	33.3 ± 6.5	35.1 ± 12.8	33 ± 11	40 (35−54)
LVEDV (ml)	194.4 ± 69.2	136.2 ± 37.4	182.3 ± 82.6	N/A	153.4 (116.5–198.0)
NT-proBNP (pg/ml)	5,174.3 ± 6,566.6	3,407 (1,948–6,790)	N/A	N/A	N/A

MitraClip device by Abbott Laboratories; Cardioband by Edwards Lifesciences, Irvine, California, USA.

HF, heart failure; LVEDD, left ventricular end diastolic diameter, LVEF, left ventricular ejection fraction, M-TEER, mitral transcatheter edge-to-edge repair.

The EuroSMR registry for over 1,000 patients with SMR and HFrEF (baseline LVEF: 35.1 ± 12.8%; other baseline HF characteristics see Table 1) reported 1-year and 2-year mortality rates after M-TEER of 20% and 32%, respectively (43). In the registry by the Italian Society of Interventional Cardiology (GISE) on the transcatheter treatment of mitral valve regurgitation (GIOTTO registry) for the cohort with SMR [*n* = 986, baseline LVEF: 32 (27–40)] following M-TEER, all-cause mortality at 1 year and 2 years was 19.0% and 30.8%, while HF hospitalization rates were 15.7% and 25.9%, respectively (44).

For MV repair using annuloplasty with the Cardioband system (Edwards Lifesciences, Irvine, California, USA) in an SMR and HFrEF population (baseline LVEF: 33 ± 11%; other baseline HF characteristics see Table 1), 1-year survival rates of 87% and 1-year survival rates free from HF readmission of 66% have been reported (41). The experience with replacement technologies to treat MR is still limited and mainly based on collective registries merging several different investigational devices. Interestingly, in the CHOICE-MI registry involving patients with midrange or preserved LVEF [baseline LVEF: 50.0 (38.1–60.0) %], the 1-year composite of all-cause mortality or HF hospitalization after transcatheter MV replacement was 39.2% (42). Similarly, the TENDER registry that collected data on patients who underwent trans-apical MV replacement using the Tendyne prosthesis (Abbott Laboratories, Chicago, Illinois, USA) reported 30-day all-cause mortality of 12%, with mean LVEF of 48 ± 12% (45).

### MR interventions in the HF disease conundrum

Following the remarkable divergence of the COAPT and the Mitra-FR trial, it is only consequential that the search for predictors of favorable outcomes after M-TEER is based on the quest for any potential explanatory discrepancy between these two trials. The concept of proportionate and disproportionate MR, namely a large coaptation defect (effective regurgitant orifice area >0.3 cm^2^) sitting over a still not too much dilated left ventricle (LVEDV index <96 ml/m^2^) as a predictor of ideal treatment response, seemed intriguing (16). However, following the positive reception of this framework, it failed to prove external validity in other M-TEER cohorts beyond the two trials it was derived from (43, 46). Based on the multi-center EuroSMR registry, Koell et al. stratified M-TEER patients per COAPT trial inclusion criteria and found that the COAPT-eligible sub-group, indeed, showed significantly lower mortality (40). Interestingly, via this stratification, they identified a sub-group of patients with preserved RV function, less TR, lower systolic pulmonary artery pressures (SPAP), and lower NT-proBNP, suggesting an earlier stage in the HF disease course. However, COAPT-ineligible patients experienced a symptomatic benefit following the M-TEER procedure. Also, a stratification of EuroSMR patients per EROA < vs. ≥0.3 cm^2^ could not add any predictive value (47). Thus, the recommendation given by the 2021 ESC/EACTS guidelines on the management of valvular heart disease seems very reasonable. In patients who meet the criteria, suggesting an increased chance of response to M-TEER, (as per Supplementary Table S7 of the guidelines these criteria are following the COAPT criteria: LVEF 20%–50%, LVESD ≤70 mm, SPAP ≤70 mmHg, absence of hemodynamic instability, and moderate or severe RV dysfunction), IIaB recommendation for M-TEER is given. However, in patients not meeting these criteria at a level, IIbC recommendation M-TEER can be performed for symptom improvement after a careful evaluation of other alternatives such as left ventricular assist device implantation or heart transplant (48). As in the COAPT trial, the exact definition of right ventricular failure is not stated and the guidelines do not give an exact cut-off; however, the value of <15 mm for tricuspid annular systolic excursion (TAPSE), based on previous literature, seems very reasonable (40).

Apart from these cardiac parameters, it is likely important to also take a more holistic perspective on the systemic status of HF patients who at the end stages of the disease may suffer from multi-organic failure (49). In line with the findings by Goliasch et al. that MR, especially, in mid-range HF has an independent negative predictive impact, it might very well be that HF patients with mid-range LVEF derive most benefits from valvular interventions. Conversely, in end-stage severe chronic HF, where the multi-organic systemic disease is the main and predominant driver of mortality, valvular intervention might be futile (31).

Additionally, not only left-sided but also right-sided HF may impact outcomes after M-TEER. In SMR patients undergoing M-TEER, Karam et al. identified right ventricular dysfunction (defined as impaired right-ventricular-to-pulmonary artery coupling, i.e., a TAPSE/sPAP ratio ≤0.274 mm/mmHg) as a significant predictor of increased 2-year mortality (50). Thus, while only left-sided interventions are being planned. Therefore, it is important to note that an additional assessment of right ventricular parameters seems to be crucial.

**Figure 2:**
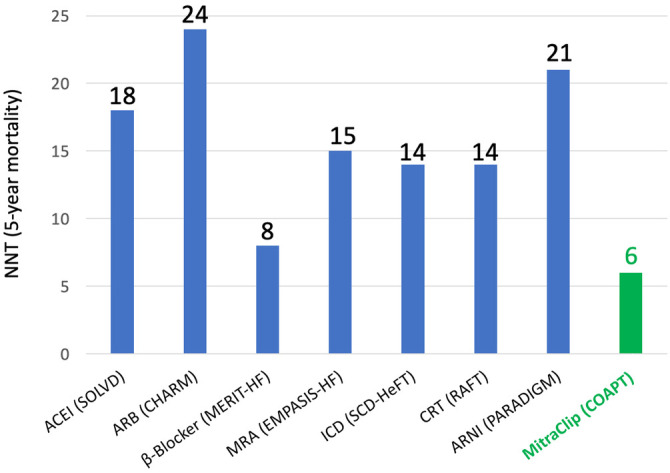
The number needed to treat (NNT) to prevent one mortality for established heart failure medications in comparison to MitraClip based on data from respective landmark trials of heart failure medications and data from the COAPT trial. Adapted from Pfister et al. (51).

Another important aspect when placing M-TEER intervention in the context of HF is GDMT and its optimization. As the pre-procedural optimization of GDMT has been a crucial part of the trial, when aiming to achieve COAPT-like results, it is a prerequisite to ensure optimized GDMT before discussing M-TEER or other transcatheter treatments. On the other hand, it is important to highlight that M-TEER in the COAPT trial showed a number needed to treat (NNT) that is lower than those of almost any HF medication or intervention (Figure 2) (51). Based on published data from respective landmark trials (SOLVD, Group M-HS, EMPHASIS-HF, SCD-HeFT, RAFT, CHARM, and PARADIGM-HF), with the assumption that all-cause mortality rates and treatment effects were constant after trial conclusion, Srivastava et al. estimated NNTs to prevent one patient from dying from several HF medications, and for all of them, they found numbers higher than the NNT of M-TEER based on COAPT data (52). Unfortunately, while in interventional trials, HF medication is assessed very rigorously in landmark HF trials; the incidence and the course of MR—and TR—are often underreported (53, 54). Thus, it is challenging to estimate each and any exact interconnection; however, it is likely key to identify the ideal interplay be it timing, dosing, or a combination of both between medical and interventional MR treatment.

Indeed, a recent study by Higuchi et al. based on the EuroSMR registry of SMR patients who underwent M-TEER was able to highlight the beneficial effects of maximized GDMT at the baseline of M-TEER and during subsequent follow-up (55). In patients who received triple GDMT (including beta-blockers, renin-angiotensin system inhibitors, and mineralocorticoid receptor antagonists), 2-year survival was higher than in those who did receive less than three GDMT drugs. The beneficial effect was confirmed, especially, in patients with kidney disease and right heart failure, and also in patients who did not have an optimal technical result after M-TEER (i.e., residual MR ≥ 2+). This, again, highlights the complementary role of M-TEER and GDMT in the complex clinical setting of HF.

## Tricuspid regurgitation

### TR in the context of heart failure

Tricuspid regurgitation has historically been considered a subsequent consequence of left-sided heart disease, and following this conception, no dedicated treatment was recommended, believing that TR would vanish after successful treatment of the left-heart disease (25). However, recently emerging evidence has proved that moderate or severe TR represents a significant predictor of mortality, independent of SPAP or LVEF (1, 56, 57). This is of high relevance as, according to the Framingham Heart Study, the incidence of TR increases with age, and severe TR is present in over 5% of women and up to 2% of men aged ≥70 years (58). A recent analysis evaluating almost 1 Million echocardiography reports from 35 community and academic cardiology centers in the US even found TR to be the most common valvular heart disease present in 7% of the overall population (median age 68 years) and up to 14% of patients ≥75 years (59). Only 8 to 10% of patients suffer from primary TR, while the vast majority of patients present with STR (60). STR may be associated with the left-sided disease even after surgical correction thereof driven by further aging, being a woman, and the presence of atrial fibrillation (61). Apart from that, STR can arise from chronic pulmonary hypertension (SPAP ≥50 mmHg) characterized by less annular dilation but severe tenting driven by long right ventricles (RVs) with elliptical/spherical deformation (62).

Koelling et al. in an HFrEF cohort (LVEF ≤ 35%) identified ≥ moderate TR in 34.5% of patients, with severe TR being a significant predictor of mortality in a multivariable analysis (63). Similar to the findings for MR by Goliasch et al., Neuhold et al. in an analysis of almost 600 patients with chronic HF identified severe TR as a significant predictor in patients with mildly or moderately impaired LVEF or with NT-proBNP levels below the median (≤280 fmol/ml) but not in those with severely impaired LVEF or with NT-proBNP levels above the median (31, 64).

### Outcomes of transcatheter treatment of TR in HF patients

The limited outcomes of TV surgery paired with the high prevalence of relevant symptomatic disease historically led to large undertreatment of TR; for example, in the US, out of the 1.6 million patients with ≥ moderate TR, less than 8,000 per year undergo surgery, resulting in a large unmet clinical need (24, 65). In-hospital mortality of isolated tricuspid valve surgery with rates of approximately 10% remains high, which is why the dedicated TRI-SCORE was developed to further stratify these high-risk patients and to allow for more suitable individualized patient management pathways (66). The large number of patients in need of treatment and the limited surgical outcomes led to the rapid development and early adoption of less invasive transcatheter treatment solutions.

Evaluating the role of transcatheter TR treatment as the first prospective single-arm trial of T-TEER using TriClip (Abbott Laboratories, Chicago, Illinois, USA), the TRILUMINATE trial presented 1-year outcomes in 85 patients (TAPSE [cm]: 1.44 ± 0.31, SPAP [mmHg]: 38.9 ± 16.0, LVEF [%]: 59.4 ± 8.1; other baseline HF characteristics see Table 2) (68). At the baseline, only 8% of patients had ≤ moderate TR, which improved to 71% at 1 year. Additionally, the functional status (NYHA class, 6MWT, KCCQ score) significantly improved and 1-year mortality was 7.1%. In the real world post-market bRIGHT study with the TriClip device at 30 days in 200 patients (TAPSE [cm]: 1.8 ± 0.9, SPAP [mmHg]: 38.8 ± 11.8, LVEF [%]: 55.6 ± 11.0; other baseline HF characteristics see Table 2), mortality was extremely low at 0.5% and TR was reduced by ≥1 grade in 81% of patients, leaving 70% of them with ≤ moderate TR (71). The prospective single-arm CLASP TR study tested the Pascal T-TEER device (Edwards Lifesciences, Irvine, California, USA) in a similar cohort (*n* = 65, *n* = 46 at 1-year follow-up; TAPSE [cm]: 1.53 ± 0.47, SPAP [mmHg]: 68% at ≥30, LVEF [%]: 57.4 ± 7.0; other baseline HF characteristics see Table 2) and at 1 year found 86% of patients at TR ≤ 2 (100% of patients with at least one grade TR reduction and 75% with at least two grades), with a significantly improved quality of life and 10.8% mortality (69, 70). In the TRI-REPAIR study, the Cardioband annuloplasty system was tested in the tricuspid position in 30 patients (TAPSE [cm]: 1.4 ± 0.3, SPAP [mmHg]: 35.9 ± 10.5, LVEF [%]: 57.5 ± 10.8; other baseline HF characteristics see Table 2), leading to 72% of patients with ≤ moderate TR and significant improvements in their quality of life at 2 years, while mortality was 26.7% at that point in time (72). With fewer hurdles (e.g., no risk of right ventricular outflow obstruction) compared to the mitral side, TV replacement is moving forward at a much higher pace. For the EVOQUE valve (Edwards Lifesciences, Irvine, California, USA), up to 6 months follow-up for 43 patients (for baseline characteristics see Table 2) was available, with 100% of patients being at none/trace or mild TR, 89% of them being in NYHA class I/II associated with a survival rate of 96% and a rate of patients free from HF hospitalization at 94% (73, 74).

**Table 2:** Baseline heart failure characteristics in main transcatheter tricuspid intervention studies.

	TRILUMINATE Pivotal RCT (*n* = 350) (67)	TRILUMINATE (*n* = 85) (68)	CLASP TR (*n* = 65, 46 at 1 year) (69, 70)	bRIGHT (*n* = 200) (71)	TRI-REPAIR (*n* = 30) (72)	TRISCEND (*n* = 132, 56 at 6 m) (73, 74)
Treatment	T-TEER (TriClip)	T-TEER (TriClip)	T-TEER (Pascal)	T-TEER (TriClip)	Annuloplasty (Cardioband)	Replacement (EVOQUE)
Implant success	98.8%	100%	91%	98%	100%	96.20%
Longest follow-up	1 year	1 year	1 year	30 days	2 years	6 months
Mortality last follow-up	9.4%	7.1%	10.8%	0.5%	26.7%	4%
**Baseline HF characteristics**						
NYHA class III/IV	59.4%	75%	79%	79%	83%	88%
LVEF (%)	59.3 ± 9.3	59.4 ± 8.1	57.4 ± 7.0	55.6 ± 11.0	57.5 ± 10.8	N/A
TAPSE (cm)	in 48% ≥ 1.7 cm	1.44 ± 0.31	1.53 ± 0.47	1.8 ± 0.9	1.4 ± 0.3	N/A
SPAP (mmHg)	39.7 ± 9.2	38.9 ± 16.0	in 68% ≥ 30	38.8 ± 11.8	35.9 ± 10.5	39.6 ± 10.8
RVEDD (cm)	5.0 ± 0.8	5.27 ± 0.67	3.99 ± 0.89	4.7 ± 0.9	3.8 ± 6.5	N/A
NT-proBNP (pg/ml)	382.0 ± 347.5 (BNP)	1,559.5 [1,002.5–2,278.0]	N/A	3,610 ± 5,662	2,925 ± 3,030	N/A

TriClip device by Abbott Laboratories; Pascal device by Edwards Lifesciences, Irvine, California, USA; Cardioband by Edwards Lifesciences, Irvine, California, USA; EVOQUE device by Edwards Lifesciences, Irvine, California, USA.

HF, heart failure; LVEF, left ventricular ejection fraction; RVEDD, right ventricular end diastolic diameter; SPAP, systolic pulmonary artery pressure; TAPSE, tricuspid annular plane systolic excursion; T-TEER, tricuspid transcatheter edge-to-edge repair.

Recently, the first randomized trial in the field of transcatheter treatment of TR has been published. The TRILUMINATE Pivotal trial randomized 350 patients to receive either T-TEER or optimized medical treatment only, with the combined primary endpoint being in favor of T-TEER treatment (67). This result mainly was driven by a marked improvement in quality of life according to the change in KCCQ score, while the other primary endpoint components mortality or TV surgery and heart failure hospitalization after a 1-year follow-up did not differ between groups. The extent of quality of life improvement was directly linked to the extent of achieved TR reduction, likely reflecting the effectiveness of the treatment. T-TEER proved to be exceptionally safe with a 30-day cardiovascular mortality of only 0.6%. While the patients according to their baseline KCCQ scores had a notably bad quality of life, the event rates for mortality and heart failure hospitalization in both groups were markedly lower than what has been observed in studies on HF patients receiving left-sided interventions, suggesting that the impact of the valvular disease on such endpoints does differ between MR and TR patients. Furthermore, the enrolled patients seem to represent a particular subset of TR patients, who mainly suffered from isolated TR, LVEF, pulmonary pressures, and pulmonary vascular resistance and were largely free from left-sided disease or pulmonary hypertension. Longer follow-up of the trial and additional studies on different patient populations will further inform the longer-term impact of T-TEER on hard endpoints and will help to identify ideal candidates for therapy.

Additional dedicated trials have started enrollment and are already close to their primary completion date (see Table 3).

**Table 3:** Ongoing randomized controlled trials evaluating transcatheter treatment of tricuspid regurgitation.

	TRILUMINATE Pivotal (NCT03904147)	TRI-FR (NCT04646811)	CLASP II TR Pivotal (NCT04097145)	TRICI-HF (NCT04634266)
Device	TriClip (T-TEER)	TriClip (T-TEER)	Pascal (T-TEER)	TriClip, Pascal (each T-TEER)
Design	RCT; vs. GDMT	RCT; vs. GDMT	RCT; vs. GDMT	RCT; vs. GDMT
Estimated enrollment (*n*)	700	300	825	360
Primary completion date	August 2022 [first results published (67)]	August 2025	December 2024	December 2025
Primary endpoint	Hierarchical composite all-cause mortality, TV surgery, HF hospitalizations, QoL with KCCQ	Milton Packer clinical composite score	Composite of all-cause mortality, RVAD implantation or heart transplant, TV intervention, HF hospitalizations, QoL by KCCQ	All-cause mortality or HF hospitalization
*HF inclusion/exclusion criteria*	Exclusion criteria: SPAP >70 mmHg or fixed pre-capillary PHT by RHC; LVEF ≤ 20%	Exclusion criteria: Uncontrolled pre-capillary PHT (RHC required), SPAP >60 mmHg; LVEF ≤ 35%	Exclusion criteria: Refractory HF requiring advanced intervention (i.e. has or will need LVAD or transplantation), ACC/AHA Stage D HF	Exclusion criteria: RHC with SPAP >70 mmHg or substantial pre-capillary PHT (mean PAP >30 mmHg plus transpulmonary gradient >17 mmHg or pulmonary vascular resistance >5 wood units)

TriClip device by Abbott Laboratories; Pascal device by Edwards Lifesciences, Irvine, California, USA.

GDMT, guideline directe medical therapy; HF, heart failure; LVAD, left ventricular assist device; LVEF, left ventricular ejection fraction; PHT, pulmonary hypertension; QoL, quality of life; RCT, randomized controlled trial; RHC, right heart catheterization; RVAD, right ventricular assist device; SPAP, systolic pulmonary artery pressure; T-TEER, tricuspid transcatheter edge-to-edge repair; TV, tricuspid valve.

### TR interventions in the setting of (right-sided) HF

Patients currently undergoing treatment are referred at the late stages of their disease as, previously, there were no treatment options available to address their persistent symptoms (75). Even though propensity-matching analyses transcatheter TR treatment could reduce mortality and HF hospitalizations in comparison to GDMT alone, the benefit seen in the randomized TRILUMINATE trial and other currently performed single-arm studies is mostly related to the quality of life measures (26, 76). Given these soft endpoints, as well as often small treatment effects, it is challenging to identify precise predictors of treatment response for the broader population based on such very selected cohorts (26, 76).

However, some first parameters potentially predicting treatment response could be identified. In general, while SMR populations present with HFrEF, in STR, LVEF is often preserved or only mildly reduced. Explanatory concepts evaluated that MR on the left side, such as a disproportionate degree of regurgitation, cannot simply be translated to the right side. For a response to interventional correction of TR, the interaction of the ventricle and the pulmonary vasculature seems to be of high relevance. Patients undergoing T-TEER showed significantly higher survival when mean (m) PAP was ≤30 mmHg and when the trans-pulmonary gradient (TPG) was ≤17 mmHg (77). If mPAP was >30 mmHg but TPG still was ≤17 mmHg (post-capillary pulmonary hypertension), treatment response was still good, but when mPAP was >30 mmHg and TPG >17 mmHg (pre-capillary hypertension), mortality after the intervention was significantly increased. This highlights the mandatory role of right heart catheterization in the work-up and evaluation of patients with STR screened for transcatheter treatment.

Not only the pulmonary vasculature itself is of predictive importance as the coupling between the right ventricle (RV) and the pulmonary arterial (PA) system can also bear prognostic implications. RV-PA coupling can be assessed as the ratio of TAPSE and SPAP, representing the contractile response of the RV to increased afterload, with lower ratios implying insufficient RV response. In the TriValve registry, when divided per TAPSE/SPAP ratio >0.406 vs. ≤0.406, patients with a lower rate of RV-PA coupling had a significantly higher risk of post-procedural mortality (78).

Of note, when assessing SPAP via echocardiography, the estimated values might differ from what would be measured invasively. Lurz et al. demonstrated that patients who echocardiographically present without pulmonary hypertension but then discordantly show pulmonary hypertension when measured invasively (pulmonary hypertension defined as SPAP ≥50 mmHg; discordant diagnosis considered when estimated SPAP differed >10 mmHg from invasive measurement) have a significantly worse prognosis (death, HF rehospitalization, and reintervention) after T-TEER (79).

In all of this, it is important to consider that most of these evaluations have been based on patient collectives that predominantly underwent T-TEER. Especially in the case of TV replacement, the role of the RV after intervention might substantially differ; as with abolished TR, the ventricle faces a substantial after-load increase that might lead to failure of the RV even though it may be only temporary.

TR patients often present even later in their disease course than those suffering from MR; thus, apart from cardiac parameters, it is important to holistically assess the status of the patient. A chronic TR state might lead to complex hypercirculatory HF impacting hepatic, renal, and intestinal function. Even though a prognostic benefit of treatment might be possible, it may be smaller among patients with chronic right HF, who show advanced congestive hepatopathy, decreased peripheral vascular tone, and potentially lack the ability to respond with venous pressures to TR reduction (80).

### Multi-valvular disease

One specific additional aspect to consider in the treatment planning of MR and/or TR might arise in the case of multi-valvular disease. The EURObservational Research Programme Valvular Heart Disease II Survey found that among over 5,000 patients with valvular heart disease, over 20% suffered from more than one valvular lesion (81). For surgical intervention, Gammie et al. recently evaluated the prognostic value of tricuspid annuloplasty performed during MV surgery whenever ≤ moderate TR was present (82). While the endpoint of less TR progression was met, this came at the cost of an increased rate of pacemaker implantations necessary in those who received TV annuloplasty, and, thus, at 2 years, no clinical benefit of such a combined approach could be demonstrated. Less invasive transcatheter treatment options, however, bring the intriguing opportunity to intervene at one valve, then wait and reevaluate other valvular lesions after a certain follow-up, and then decide whether an additional procedure is really needed (83).

### Future perspectives

It is obvious that transcatheter MR and TR interventions are addressing a complex disease conundrum often characterized by chronic HF; thus, a simple, standardized, and straightforward treatment algorithm, for example in aortic stenosis, does not likely exist.

To allow transcatheter MR and TR interventions to fully exploit the potential they bear for HF patients, a paradigm shift regarding the intended role of these procedures might be needed. Only when such interventions are considered synergistic with HF medications and, thus, are included in the discussion of treatment options along the whole course of progressing HF, they can then be applied at that exact point of the disease course when they will be most beneficial. However, if these transcatheter interventions continue to be only considered bailouts when GDMT has been fully optimized and failed to optimally control HF symptoms, they will often be likely applied after the occurrence of irreversible changes to cardiac structures and other organs that otherwise could have been prevented. An open and cooperative heart team, including sub-specialties such as HF experts, clinical cardiologists, and geriatricians, is the ideal platform for such discussion and at the same time represents the key prerequisite to establishing a future-oriented HF treatment armamentarium, including transcatheter MR and TR interventions.

It has become evident that HF cannot be sufficiently characterized by only one cut-off value, namely LVEF, which itself is an often dynamic parameter and at times imprecise. A more distinct characterization of HF must include several different cardiac parameters as well as a holistic appreciation of the organic status in elderly patients. Assessing cardiac structures must incorporate a broad appreciation of the ventricular-annular unit, including assessment of LV dimensions, pressure and contractility, annular dimensions and contractility, as well as synchrony and synergy of the whole atrio-annular-ventricular valve apparatus with its impact on coaptation and tethering (84). Here, utilizing new technologies such as machine learning approaches, scanning already existing multi-parametric data, new phenotypes of HF, and structural heart alterations that might remain hidden with conventional methods, could be identified (85). When assessing HF from a more holistic perspective, that also appreciates other organs apart from the heart itself, a realistic and self-critical appraisal is warranted, considering what, given such a multi-morbid complex late-stage disease setting, might be the remaining potential of an intervention addressing only the cardiac structure.

## Conclusions

As patients with severe symptomatic SMR and STR are often suffering from chronic HF, evaluating such patients for treatment and finally performing transcatheter interventions in such a condition poses a challenge for inter-disciplinary heart teams. Following the growing experience, especially with TEER, the first markers of likely treatment response could be identified. In SMR, patients should match the COAPT trial criteria, as then an actual prognostic benefit from intervention can be drawn. However, also in COAPT-ineligible patients, intervention should be discussed as a substantial alleviation of symptoms is still achievable for them. In STR patients, an RHC should be performed when evaluating potential treatment candidates, and pre-capillary pulmonary hypertension should be excluded before interventional treatment of TR.

Finally, in the future, the heart team should discuss transcatheter interventions for SMR and STR ideally as one part of a synergistic framework alongside established HF medications. In chronic HF, only a multifaceted holistic treatment approach can likely bring the potential lifesaving therapeutic effects of current medical and interventional innovations to these patients in need.

## Data Availability

The original data presented in the manuscript are derived from previously published studies that are reported in the references.
