# Supplementary material for: Transcatheter mitral and tricuspid interventions—the bigger picture: valvular disease as part of heart failure
Source: Front Cardiovasc Med. 2023 May 15;10:1091309. doi: 10.3389/fcvm.2023.1091309 (PMC10225583; doi:10.3389/fcvm.2023.1091309)
Supplement: Supplementary file 1 [file Table1.docx]

| **Table 1.** Baseline heart failure characteristics in main transcatheter mitral intervention studies. | | | | | |
| --- | --- | --- | --- | --- | --- |
|  | COAPT (n=302) (14) | Mitra.FR (n=152) (15) | EuroSMR (n=1016) (46) | Cardioband 1y (n=60) (42) | CHOICE-MI (43) |
| Treatment | M-TEER (MitraClip) | M-TEER (MitraClip) | M-TEER (MitraClip) | Annuloplasty (Cardioband) | Replacement (10 different devices) |
| Longest follow-up | 3 years | 2 years | 2 years | 1 year | 1 year |
| Mortality last follow-up | 42.8% | 63.80% | 32%% | 13%% | 28% |
| *Baseline HF characteristics* | | | | | |
| NYHA class III/IV | 57% | 63% | 89% | 87% | 87% |
| LVEF (%) | 31.3 ± 9.1 | 33.3 ± 6.5 | 35.1 ± 12.8 | 33 ±11 | 40 (35-54) |
| LVEDV (ml) | 194.4 ± 69.2 | 136.2 ± 37.4 | 182.3 ± 82.6 | N/A | 153.4 (116.5-198.0) |
| NT-proBNP (pg/ml) | 5174.3 ± 6566.6 | 3407 (1948-6790) | N/A | N/A | N/A |
| Legend: MitraClip device by Abbott Laboratories; Cardioband by Edwards Lifesciences, Irvine, California, USA. Abbreviations: HF, heart failure; LVEDD, left ventricular end diastolic diameter, LVEF, left ventricular ejection fraction, M-TEER, mitral transcatheter edge-to-edge repair. | | | | | |
|  |  |  |  |  |  |
|  |  |  |  |  |  |
|  |  |  |  |  |  |
