# Supplementary material for: Transcatheter mitral and tricuspid interventions—the bigger picture: valvular disease as part of heart failure
Source: Front Cardiovasc Med. 2023 May 15;10:1091309. doi: 10.3389/fcvm.2023.1091309 (PMC10225583; doi:10.3389/fcvm.2023.1091309)
Supplement: Supplementary file 2 [file Table2.docx]

| **Table 2.** Baseline heart failure characteristics in main transcatheter tricuspid intervention studies. | | | | | | |
| --- | --- | --- | --- | --- | --- | --- |
|  | TRILUMINATE Pivotal RCT (n=350) (74) | TRILUMINATE (n=85) (67) | CLASP TR (n=65, 46 at 1y) (69,70) | bRIGHT (n=200) (68) | TRI-REPAIR (n=30) (71) | TRISCEND (n=132, 56 at 6m) (72,73) |
| Treatment | T-TEER (TriClip) | T-TEER (TriClip) | T-TEER (Pascal) | T-TEER (TriClip) | Annuloplasty (Cardioband) | Replacement (EVOQUE) |
| Implant success | 98.8% | 100% | 91% | 98% | 100% | 96.20% |
| Longest follow-up | 1 year | 1 year | 1 year | 30 days | 2 years | 6 months |
| Mortality last follow-up | 9.4% | 7.1% | 10.8% | 0.5% | 26.7% | 4% |
| *Baseline HF characteristics* | | | | | | |
| NYHA class III/IV | 59.4% | 75% | 79% | 79% | 83% | 88% |
| LVEF (%) | 59.3 ± 9.3 | 59.4 ± 8.1 | 57.4 ± 7.0 | 55.6 ± 11.0 | 57.5 ± 10.8 | N/A |
| TAPSE (cm) | in 48% ≥ 1.7 cm | 1.44 ± 0.31 | 1.53 ± 0.47 | 1.8 ± 0.9 | 1.4 ± 0.3 | N/A |
| SPAP (mmHg) | 39.7 ± 9.2 | 38.9 ± 16.0 | in 68% ≥ 30 | 38.8 ± 11.8 | 35.9 ± 10.5 | 39.6 ± 10.8 |
| RVEDD (cm) | 5.0 ± 0.8 | 5.27 ± 0.67 | 3.99 ± 0.89 | 4.7 ± 0.9 | 3.8 ± 6.5 | N/A |
| NT-proBNP (pg/ml) | 382.0 ± 347.5 (BNP) | 1559.5 [1002.5-2278.0] | N/A | 3610 ± 5662 | 2925 ± 3030 | N/A |
| Legend: TriClip device by Abbott Laboratories; Pascal device by Edwards Lifesciences, Irvine, California, USA; Cardioband by Edwards Lifesciences, Irvine, California, USA; EVOQUE device by Edwards Lifesciences, Irvine, California, USA. Abbreviations: HF, heart failure; LVEF, left ventricular ejection fraction; RVEDD, right ventricular end diastolic diameter; SPAP, systolic pulmonary artery pressure; TAPSE, tricuspid annular plane systolic excursion; T-TEER, tricuspid transcatheter edge-to-edge repair. | | | | | | |
