# Supplementary material for: Transcatheter mitral and tricuspid interventions—the bigger picture: valvular disease as part of heart failure
Source: Front Cardiovasc Med. 2023 May 15;10:1091309. doi: 10.3389/fcvm.2023.1091309 (PMC10225583; doi:10.3389/fcvm.2023.1091309)
Supplement: Supplementary file 3 [file Table3.docx]

| **Table 3.** Ongoing randomized controlled trials evaluating transcatheter treatment of tricuspid regurgitation. | | | | |  |
| --- | --- | --- | --- | --- | --- |
|  | TRILUMINATE Pivotal (NCT03904147) | TRI-FR (NCT04646811) | CLASP II TR Pivotal (NCT04097145) | TRICI-HF (NCT04634266) |  |
| Device | TriClip (T-TEER) | TriClip (T-TEER) | Pascal (T-TEER) | TriClip, Pascal (each T-TEER) |  |
| Design | RCT; vs. GDMT | RCT; vs. GDMT | RCT; vs. GDMT | RCT; vs. GDMT |  |
| Estimated enrollment (n) | 700 | 300 | 825 | 360 |  |
| Primary completion date | August 2022 (first results published (72)) | August 2025 | December 2024 | December 2025 |  |
| Primary endpoint | Hierarchical composite all-cause mortality, TV surgery, HF hospitalizations, QoL with KCCQ | Milton Packer clinical composite score | Composite of all-cause mortality, RVAD implantation or heart transplant, TV intervention, HF hospitalizations, QoL by KCCQ | All-cause mortality or HF hospitalization |  |
| *HF inclusion/exclusion criteria* | Exclusion criteria: SPAP > 70 mmHg or fixed pre-capillary PHT by RHC; LVEF ≤ 20% | Exclusion criteria: Uncontrolled pre-capillary PHT (RHC required), SPAP > 60 mmHg; LVEF ≤ 35% | Exclusion criteria: Refractory HF requiring advanced intervention (i.e. has or will need LVAD or transplantation), ACC/AHA Stage D HF | Exclusion criteria: RHC with SPAP > 70 mmHg or substantial pre-capillary PHT (mean PAP> 30 mmHg plus transpulmonary gradient > 17 mmHg or pulmonary vascular resistance > 5 wood units) |  |
| Legend: TriClip device by Abbott Laboratories; Pascal device by Edwards Lifesciences, Irvine, California, USA. Abbreviations: GDMT, guideline directe medical therapy; HF, heart failure; LVAD, left ventricular assist device; LVEF, left ventricular ejection fraction; PHT, pulmonary hypertension; QoL, quality of life; RCT, randomized controlled trial; RHC, right heart catheterization; RVAD, right ventricular assist device; SPAP, systolic pulmonary artery pressure; T-TEER, tricuspid transcatheter edge-to-edge repair; TV, tricuspid valve | | | | |  |
|  |  |  |  |  |  |
